# Epidemiological Profile, Causes, and Trends of Mortality in the Emergency Department of a Tertiary Hospital in Northern India: A Five-Year Retrospective Study

**DOI:** 10.7759/cureus.113759

**Published:** 2026-07-31

**Authors:** Samina Mufti, Sahibzada Junaid, Irum Amin, Tuaiba Ali, Showkat Mufti

**Affiliations:** 1 Hospital Administration, Sher-i-Kashmir Institute of Medical Sciences, Srinagar, IND; 2 Obstetrics and Gynaecology, Lady Hardinge Medical College, New Delhi, IND; 3 Emergency Medicine, Sher-i-Kashmir Institute of Medical Sciences, Srinagar, IND

**Keywords:** emergency mortality, icd-10, intracranial haemorrhage, kaplan-meier, septicaemia, standardized mortality ratio, tertiary care

## Abstract

Background: Emergency departments (EDs) in tertiary care hospitals serve as the primary interface for critically ill patients and are key indicators of health system performance. Mortality data from EDs provide actionable insights for quality improvement, resource allocation, and policy planning.

Objectives: The objective of this study was to analyse patterns of emergency mortality at a tertiary care teaching hospital over five years (2021-2025) across temporal, demographic, diagnostic, and specialty-specific dimensions.

Methods: A retrospective analysis of 38,818 emergency admissions from January 2021 to December 2025 was performed. Medical records data coded with the International Classification of Diseases, Tenth Revision (ICD-10) were analysed after correction of coding errors and diagnostic reclassification. Statistical analyses included Kaplan-Meier survival analysis, chi-square tests, Mann-Whitney U and Kruskal-Wallis H tests, binary logistic regression, and the Standardized Mortality Ratio (SMR).

Results: A total of 7,318 deaths were recorded, for an overall crude mortality rate of 18.9%. Mortality declined from 26.2% in 2021 to 12.7% in 2025. Winter (23.6%), the dawn period (04:00-07:59 hours; 34.3%), and the night shift (27.3%) carried the highest mortality. Elderly patients aged 61+ years accounted for 3,879 (53.0%) deaths, at a 33.3% mortality rate. The circulatory system was the leading cause (1,954 deaths; 26.7%), while septicaemia (A41.9) was the single most common diagnosis (742 deaths). Increasing age was the strongest independent predictor of death (adjusted OR (aOR) 5.21, 95% CI 4.63-5.86). The SMR fell from 2.62 to 1.27 but remained above unity throughout.

Conclusion: The ED demonstrates significant temporal, demographic, and diagnostic mortality patterning. The declining SMR reflects meaningful quality improvement, yet persistent excess mortality during winter, at night, and among the elderly identifies clear targets for system-level intervention.

## Introduction

Emergency departments (EDs) are the frontline of acute care delivery, managing a heterogeneous burden of disease ranging from trauma and cardiovascular emergencies to infectious disease and end-stage organ failure. In low- and middle-income countries (LMICs), EDs bear a disproportionate share of critical illness attributable to delayed presentations, limited primary care access, and high-acuity referral patterns [1,2]. India’s emergency medicine landscape has evolved substantially over the past two decades; however, ED mortality remains significantly higher than in high-income countries, reflecting structural deficits in prehospital care, triage systems, and critical care capacity [3,4].

Globally, cardiovascular disease remains the leading cause of death, accounting for 17.9 million deaths annually [5]. Sepsis, the systemic response to infection, is responsible for an estimated 11 million deaths per year worldwide, with a disproportionate burden in South Asia and sub-Saharan Africa [6,7]. Stroke, encompassing both haemorrhagic and ischaemic subtypes, ranks among the leading causes of premature mortality in South Asian populations, including India, where hypertension prevalence, cold-climate effects, and limited neuroimaging availability amplify its lethality [8,9].

Mortality surveillance in emergency settings provides a unique window into population health burden, health system performance, and quality-of-care gaps. Despite this, systematic multi-year analyses of emergency mortality from Indian tertiary hospitals, particularly from northern and high-altitude regions, remain sparse in the published literature [10,11]. This study aimed to comprehensively characterise the patterns of emergency mortality over a five-year period (2021-2025) in a government tertiary care teaching hospital in northern India, incorporating year-wise, monthly, seasonal, and circadian mortality trends, demographic associations by age and sex, International Classification of Diseases, Tenth Revision (ICD-10) [12] chapter and diagnosis-level cause-of-death analysis, specialty-specific mortality profiling; Kaplan-Meier survival analysis with log-rank testing, binary logistic regression for independent predictors of mortality, and Standardised Mortality Ratio benchmarking against national emergency department mortality data.

## Materials and methods

This was a retrospective, observational, single-centre study conducted in the ED of Sher-i-Kashmir Institute of Medical Sciences (SKIMS), Soura, Srinagar, Jammu and Kashmir, India. The study hospital is situated at an altitude of approximately 1,600 metres and serves a geographically isolated, climatically extreme population in a region characterised by marked epidemiological transition and a dual burden of non-communicable and communicable diseases [13]. The ED receives referrals from across a mountainous catchment area of over 12 million people, often as last-resort tertiary referrals of critically ill patients with advanced disease, resulting in an inherently high-acuity, high-mortality patient population. The ED operates around the clock and manages a high-acuity case mix dominated by cardiovascular, respiratory, infectious, and oncological emergencies, frequently receiving critically ill patients with advanced disease who have already passed through multiple levels of care.

The study was approved by the Institutional Ethics Committee of SKIMS (approval number: SIMS 131/IEC-SKIMS/2026-210) and conducted in accordance with the principles of the Declaration of Helsinki. Because the analysis used only anonymised, routinely collected hospital records with no direct patient contact, the requirement for individual informed consent was waived; patient confidentiality was preserved throughout by de-identification of all records before analysis.

Study period and population

The study population comprised all patients admitted to an inpatient specialty through the emergency services between January 1, 2021, and December 31, 2025. Because the analysis encompassed every eligible admission across the five-year interval rather than a probability sample, it constitutes a complete enumeration (census) of the ED admission cohort. The primary outcome of interest was in-hospital death following emergency admission; both survivors and non-survivors were retained in the dataset so that mortality rates, survival distributions, and independent predictors of death could be derived from the full denominator of admissions.

Inclusion and Exclusion Criteria

All patients of any age and either sex admitted through the ED during the study period were eligible for inclusion. Records were excluded if they were duplicate entries or lacked a verifiable admission date, a recorded outcome, or the particular variable required for a given analysis. To preserve data integrity, missing values for individual stratifying variables (for example, documented sex or exact admission time) were handled by listwise deletion within the corresponding analysis; as a result, the denominators of certain cross-tabulations are marginally lower than the total eligible cohort, and these minor differences are indicated in the relevant table footnotes.

Sample Size

No a priori sample size calculation was required because the study analysed the entire eligible population rather than a sampled subset. The study therefore constituted a complete enumeration of all emergency admissions during the five-year period. For contextual benchmarking, the expected ED mortality/incidence rate used in the SMR analysis was based on the pooled ED mortality estimate reported in comparable low- and middle-income settings [14]. 

Data source and study parameters

Data were extracted from the hospital’s digitised admission and discharge records. As this was a retrospective analysis of pre-existing records, no questionnaire, survey instrument, or direct patient interview was employed at any stage; all variables were obtained from routinely collected hospital documentation. The parameters captured for each admission were age, sex, district of residence, type of emergency (medical, surgical, or paediatric), final clinical diagnosis, the corresponding ICD-10 code, admitting specialty, and the date and time of both admission and outcome.

Data quality assurance

Before analysis, the extracted records were screened for duplicate entries, missing essential variables, inconsistent admission and outcome dates, and discrepancies between recorded diagnoses and corresponding ICD-10 codes. Identified coding inconsistencies were reviewed and corrected before diagnostic classification. Prior to analysis, the records underwent systematic correction of coding errors and diagnostic reclassification to align with standard ICD-10 cause-of-death conventions, ensuring that each death was assigned to a single, clinically appropriate underlying cause. Records lacking a verifiable admission date or documented outcome were excluded. Missing values for individual variables were handled through analysis-specific listwise deletion, and the applicable denominators are reported in the relevant tables.

Operational definitions

Patients were grouped into predefined age strata (<1, 1-4, 5-17, 18-40, 41-60, and 61+ years). Seasons were defined according to the regional climate as winter (December-February), spring (March-May), summer (June-August), and autumn (September-November). The 24-hour clock was partitioned both into six-hour circadian blocks and into three nursing shifts: morning (08:00-16:00 hours), evening (16:00-24:00 hours), and night (00:00-08:00 hours). Early mortality was defined as death within 24 hours of admission, and time-to-death was categorised as <6, 6-24, 24-48, and >48 hours. Length of stay (LOS) was measured in days, and final diagnoses were aggregated into major ICD-10 chapters for cause-of-death analysis.

Statistical analysis

Descriptive statistics summarised demographic and clinical characteristics. Categorical variables were expressed as frequencies and percentages in the format n (%); continuous variables were summarised using means with standard deviations (SD) or medians with interquartile ranges (IQR), depending on data distribution. Associations between categorical variables and mortality were assessed using the Pearson chi-square (χ²) test, or Fisher’s exact test where expected cell counts were small, with Cramér’s V reported as a measure of effect size. Differences in length of stay were examined using the Mann-Whitney U test for two-group comparisons and the Kruskal-Wallis H test for comparisons across three or more groups. Survival time from admission to death was analysed using the Kaplan-Meier method, and survival distributions across strata were compared using the log-rank test. Binary logistic regression was performed to identify independent predictors of mortality; variables demonstrating clinical relevance or statistical significance in univariate analysis were entered into the multivariable model, and adjusted odds ratios (aORs) with 95% confidence intervals (CIs) were reported. Model performance was assessed using the Nagelkerke R² and the Hosmer-Lemeshow goodness-of-fit test, and multicollinearity was evaluated before final model construction. The SMR was calculated as the ratio of observed deaths to expected deaths, with expected deaths derived by applying a conservative reference ED mortality of 10% [14] to the annual admission count; 95% CIs for the SMR were computed assuming a Poisson distribution of observed deaths. Analysis was performed using R version 4.3.3 (R Foundation for Statistical Computing, Vienna, Austria) and IBM SPSS Statistics for Windows, version 26 (IBM Corp., Armonk, New York, United States), with a two-sided p-value <0.05 considered statistically significant.

## Results

Overview of emergency admissions and mortality

**Figure 1:**
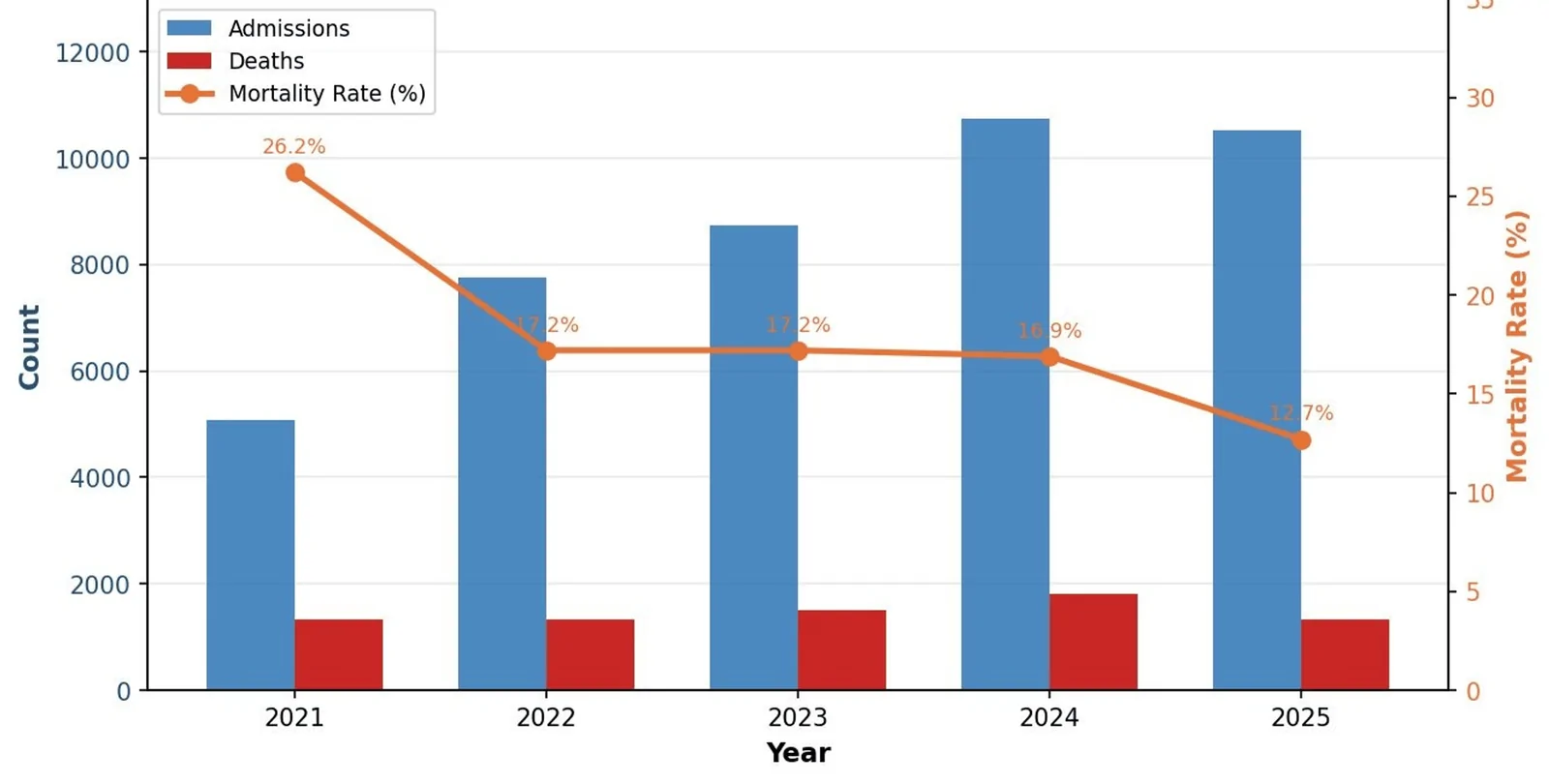
Five-year emergency admissions, deaths, and mortality rate (2021–2025). The mortality rate fell from 26.2% in 2021 to 12.7% in 2025.

Across the five-year period, a total of 38,818 emergency admissions yielded 7,318 deaths, corresponding to an overall crude mortality rate of 18.9%. The annual mortality rate declined progressively from 26.2% in 2021 to 12.7% in 2025, a near-halving over the study interval (Figure 1). This downward trajectory was steepest immediately after 2021, the peak pandemic year, and then continued more gradually through to 2025.

Temporal patterns: monthly distribution

**Figure 2:**
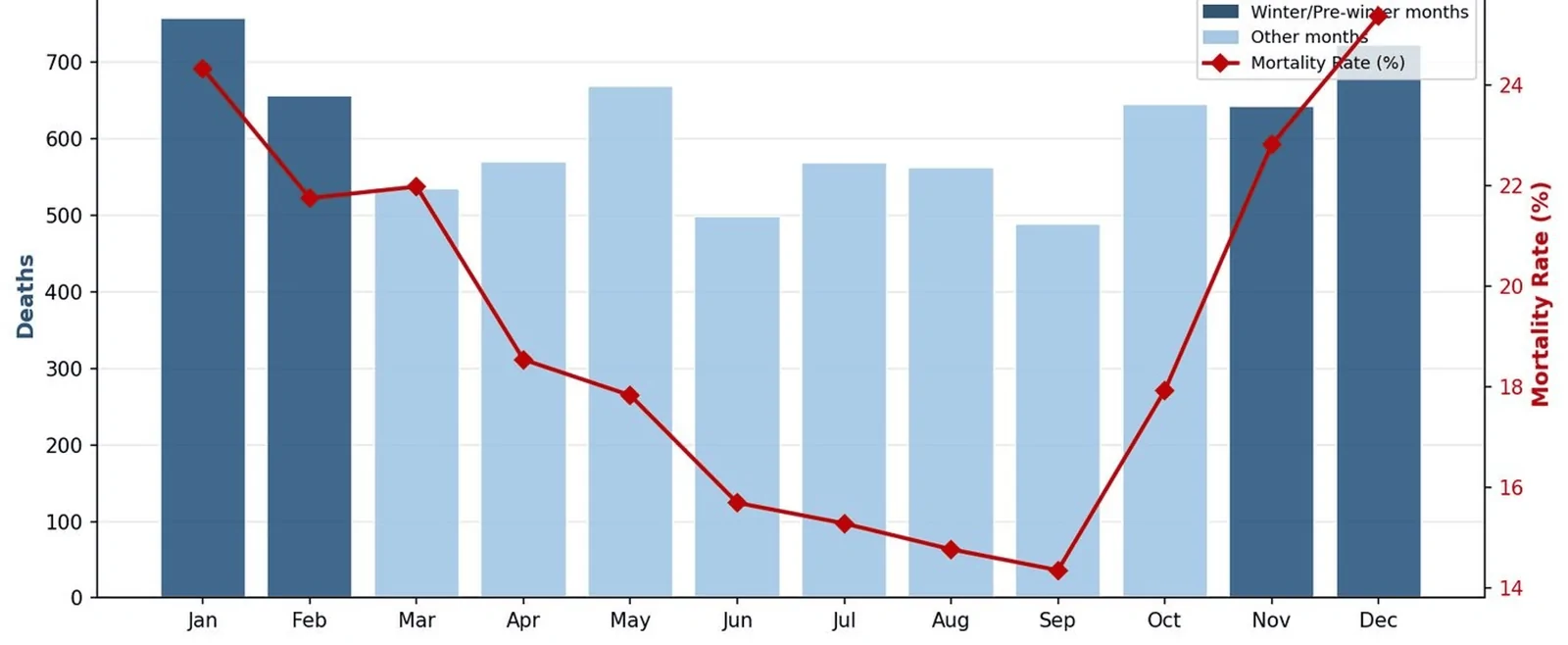
Monthly emergency deaths and mortality rate (pooled 2021–2025). Winter months (December–February) and the pre-winter rise in November show elevated mortality relative to summer.

Monthly mortality demonstrated a clear seasonal structure, peaking in the winter months of December (25.4%) and January (24.3%) and reaching its nadir in late summer, in September (14.4%) and August (14.8%) (Figure 2). A consistent pre-winter rise was evident from November onward, mirroring the seasonal cardiovascular and respiratory disease burden of the region. The amplitude between the highest and lowest months exceeded ten percentage points, underscoring the magnitude of seasonal patterning in emergency mortality.

Temporal patterns: seasonal distribution

When pooled by season, winter carried both the highest mortality rate (23.6%) and the largest share of deaths (2,124 deaths; 29.0% of all deaths), despite contributing fewer admissions than summer (Table 1). Summer recorded the lowest mortality rate (15.6%), even though it accounted for the greatest admission volume (10,712 admissions). The gradient from winter through spring (19.0%) and autumn (18.0%) to summer was monotonic, and the absolute winter excess of approximately eight percentage points over summer translated into several hundred additional deaths concentrated in the coldest quarter.

**Table 1:** Seasonal distribution of emergency admissions, deaths, and mortality rate (pooled 2021–2025). The total reflects the 38,772 admissions with complete admission-date data; 46 records (0.1% of the full cohort of 38,818) with a missing or unverifiable admission date were excluded from temporal analyses. Deaths (n = 7,318) were documented in all records. Winter (December–February) is defined per the regional climate. Mortality rate = deaths / admissions × 100.

Season	Admissions, n	Deaths, n	Mortality Rate, %	Percentage of Deaths
Winter (December–February)	8,983	2,124	23.6%	29.0%
Spring (March–May)	9,260	1,763	19.0%	24.1%
Autumn (September–November)	9,817	1,765	18.0%	24.1%
Summer (June–Aug)ust	10,712	1,666	15.6%	22.8%
TOTAL	38,772	7,318	18.9%	100%

Temporal patterns: hour-of-day analysis

**Figure 3:**
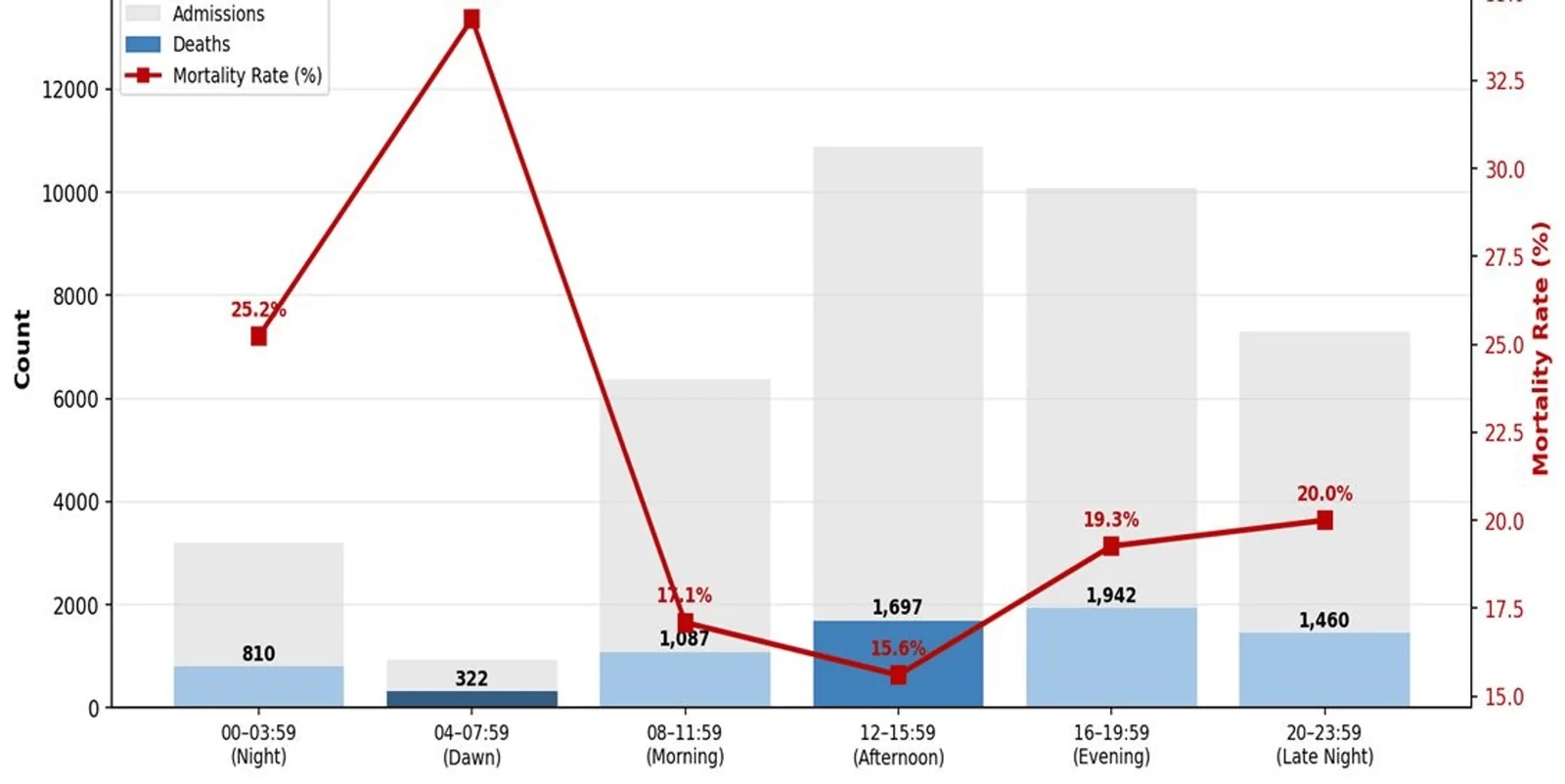
Hour-of-day analysis of emergency admissions and deaths (six-hour blocks). The dawn period (04:00–07:59 hours) shows the highest mortality rate despite the lowest admission volume.

Circadian analysis revealed that the dawn period (04:00-07:59 hours) carried the highest mortality rate (34.3%) despite recording the lowest admission volume of any six-hour block, whereas the afternoon block (12:00-15:59 hours) had the lowest mortality rate (Figure 3). This inverse relationship between admission volume and lethality indicates that the dawn excess is driven by case severity rather than by departmental crowding. The pattern is consistent with the early-morning chronobiology of acute cardiovascular and cerebrovascular events and coincides with the period of lowest staffing density in the department.

Temporal patterns: shift analysis

Shift-level analysis confirmed the nocturnal excess: the night shift (00:00-08:00 hours) recorded the highest mortality rate (27.3%) and accounted for a disproportionate share of deaths (1,132 deaths; 15.5%) relative to its admission share (4,150 admissions; 10.7%), yielding a positive net-shift differential of +4.8% (Table 2). By contrast, the morning shift carried a negative differential (−6.5%), contributing proportionally fewer deaths (2,784; 38.0%) than admissions (17,240; 44.5%), while the evening shift occupied an intermediate position (+1.7%). These differentials indicate that mortality is concentrated precisely in the hours when admission volume is lowest, reinforcing the dawn-period finding and pointing to a system-level vulnerability during overnight operation.

**Table 2:** Shift analysis of emergency admissions, deaths, and mortality rate. Net shift = death share minus admission share. The total reflects the 38,772 admissions with a documented admission time; the 46 records with missing admission time were excluded from this analysis. A positive net-shift value indicates that the shift accounts for a greater proportion of deaths than of admissions.

Shift	Admissions (n=38,772), n (%)	Deaths (n=7,318), n (%)	Mortality Rate	Net Shift
Morning (08:00–16:00)	17,240 (44.5%)	2,784 (38.0%)	16.1%	−6.5%
Evening (16:00–24:00)	17,382 (44.8%)	3,402 (46.5%)	19.6%	+1.7%
Night (00:00–08:00)	4,150 (10.7%)	1,132 (15.5%)	27.3%	+4.8%

Patient demographics: age group

**Figure 4:**
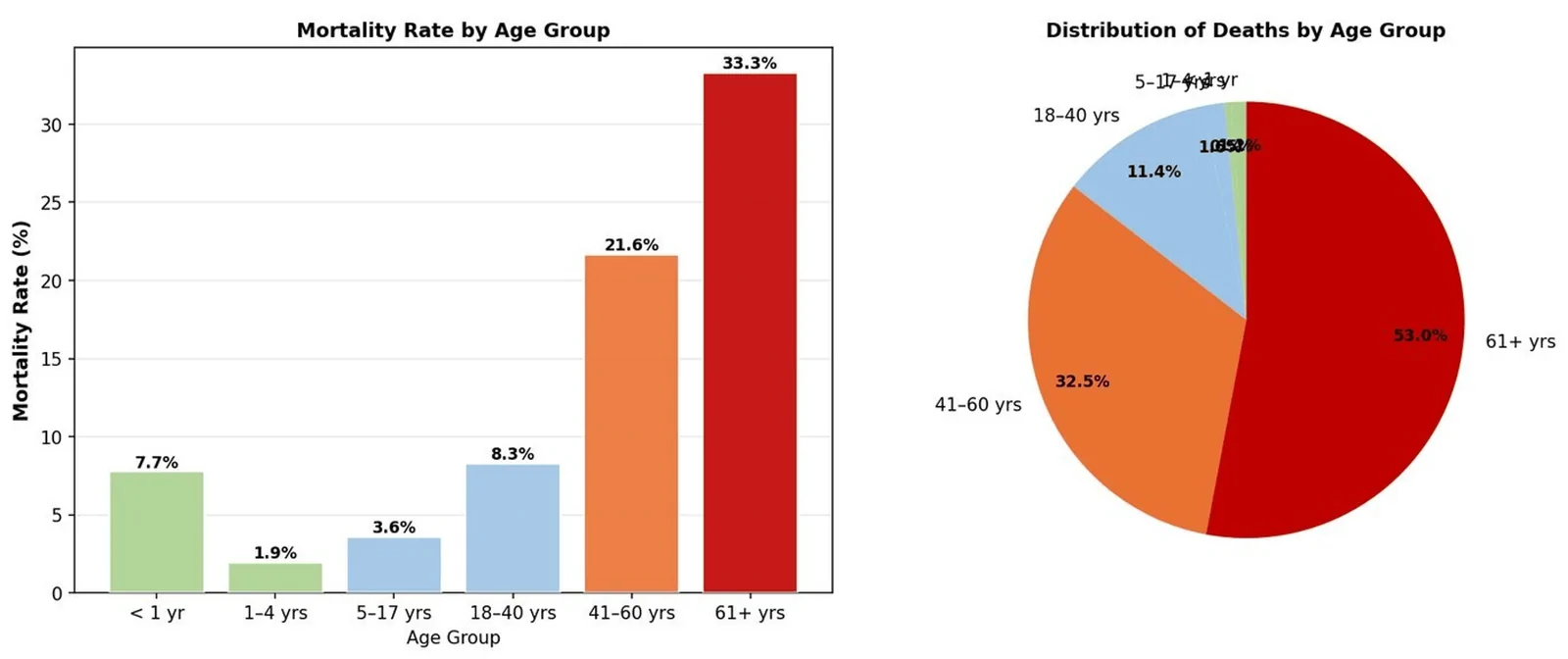
Age-group analysis of emergency mortality (five-year pooled). Mortality increases monotonically with age, peaking at 33.3% in patients aged 61+ years.

Mortality rose steeply and monotonically with advancing age (Figure 4). Elderly patients aged 61 years and above experienced a 33.3% mortality rate and accounted for more than half of all deaths (3,879 deaths; 53.0%), such that approximately one in three elderly emergency admissions ended in death. In stark contrast, children aged 1-4 years had the lowest mortality rate (1.9%), with rates climbing steadily across each successive adult stratum. This concentration of mortality in the oldest group reflects the compounding effect of frailty and multimorbidity on acute illness trajectories and identifies the elderly as the single largest contributor to the institutional death burden.

Patient demographics: sex distribution

Although male patients contributed more absolute deaths (4,171 deaths; 57.0%), female patients demonstrated a higher crude mortality rate (19.7% versus 18.3% in males) (Table 3). Female patients accounted for 3,147 deaths (43.0%) from a smaller admission base of 15,950, whereas male patients contributed 4,171 deaths from 22,782 admissions. This apparent paradox, fewer female deaths in absolute terms yet a higher female case-fatality rate, persisted after multivariable adjustment and may reflect delayed care-seeking, more advanced disease at presentation, and sex-specific physiological responses to critical illness among women

**Table 3:** Sex distribution of emergency admissions, deaths, and mortality rate (five-year pooled). The total reflects the 38,732 admissions with documented sex; 86 records (0.2% of the full cohort of 38,818) with undocumented sex were excluded from this analysis Female patients showed a higher mortality rate despite fewer absolute deaths, a differential that persisted after multivariable adjustment.

Sex	Admissions	Deaths	Mortality Rate	% of All Deaths	Remark
Male	22,782	4,171	18.3%	57.0%	Higher absolute deaths
Female	15,950	3,147	19.7%	43.0%	Higher mortality rate
TOTAL	38,732	7,318	18.9%	100%	—

Clinical outcome: LOS before death

The temporal profile of death was hyper-acute, with the great majority of deaths occurring soon after arrival (Table 4). A total of 6,096 deaths (83.3%) occurred within the first 72 hours of admission, against a mean length of stay of 2.4 days, while only 1,222 deaths (16.7%) occurred beyond 72 hours. This predominance of very early death is characteristic of a last-resort tertiary referral department that receives patients who are already critically ill on arrival or who deteriorate rapidly, whereas the smaller late-death fraction may partly reflect prolonged boarding while awaiting ward transfer.

**Table 4:** Length of stay before death in the emergency department. The predominance of deaths within 72 hours reflects the high-acuity, last-resort referral character of the department. Counts are expressed as n, percentages and mortality rates as %, and length of stay is expressed in days where applicable.

Stay Duration	Deaths	% of Total	Clinical Interpretation
1–3 days (≤ 72 h)	6,096	83.3%	May be Critically ill on arrival / rapid deterioration
> 3 days (> 72 h)	1,222	16.7%	Over boarding / awaiting ward transfer
TOTAL	7,318	100%	Mean LOS = 2.4 days

ICD-10 cause-of-death analysis

**Figure 5:**
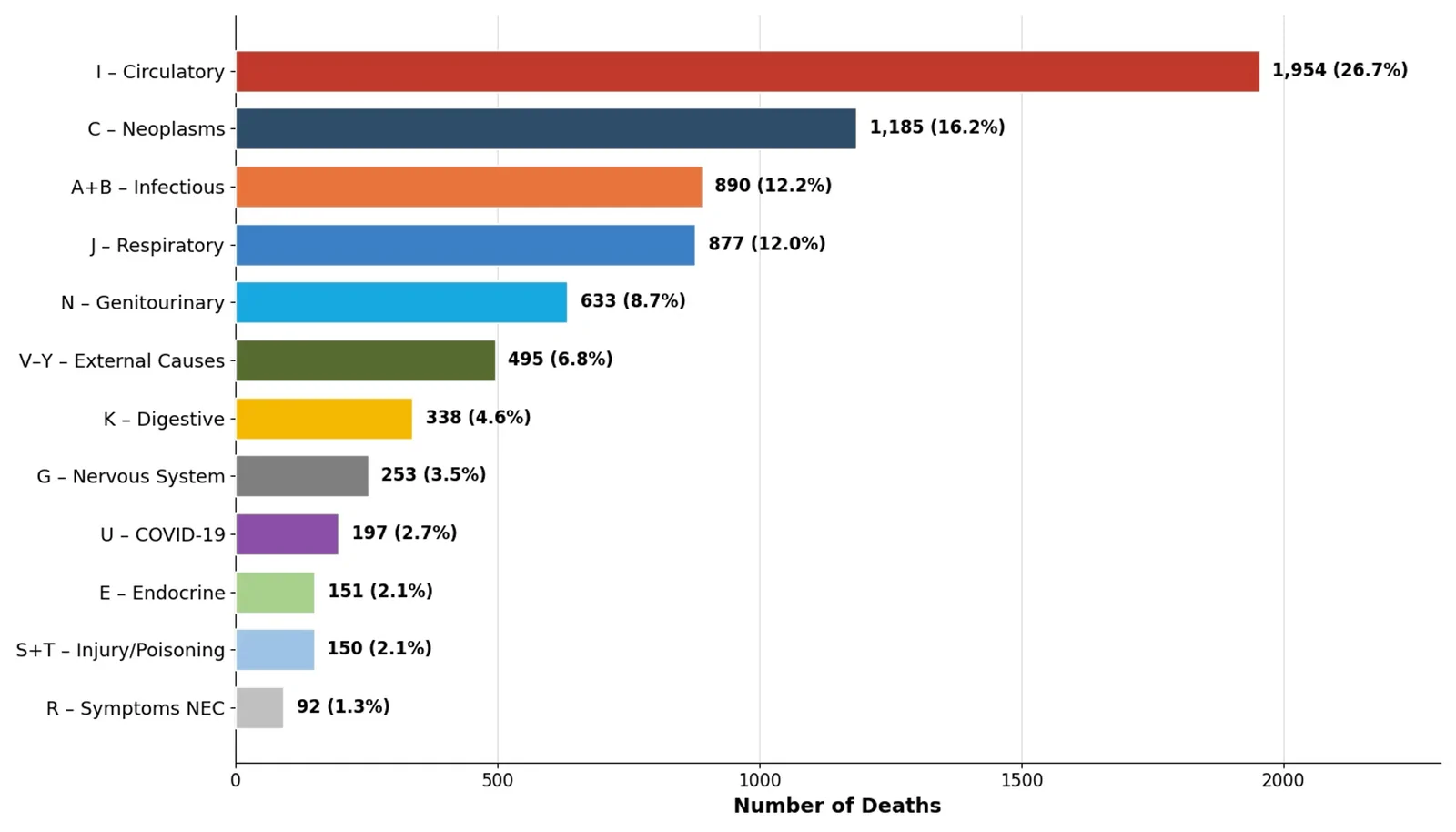
ICD-10 chapter-wise mortality distribution (total deaths = 7,318). The circulatory system dominates, followed by neoplasms and infectious causes.

The circulatory system (ICD-10 Chapter I) was the leading cause of emergency death, accounting for 1,954 deaths (26.7%), followed by neoplasms (1,185 deaths; 16.2%) and infectious and parasitic diseases (890 deaths; 12.2%) (Figure 5, Table 5). Respiratory (877 deaths; 12.0%) and genitourinary (633 deaths; 8.7%) causes followed, while the remaining chapters each contributed smaller proportions. Coronavirus disease 2019 (COVID-19) was distinguished by the longest mean LOS of any chapter (7.1 days), with 70% of its deaths occurring beyond 48 hours, a protracted survival-to-death trajectory that contrasted sharply with the rapid deaths seen in circulatory disease, of which 68.2% occurred within 24 hours. This divergence in time-to-death across chapters illustrates that different disease processes impose distinct temporal demands on emergency and critical-care resources.

**Table 5:** ICD-10 chapter-wise mortality summary, with mean length of stay and time-to-death distribution. The 12 chapters listed account for 7,215 of the 7,318 deaths (98.6%); the remaining 103 deaths (1.4%) occurred in chapters each contributing fewer than 90 deaths (including haematological, mental and behavioural, musculoskeletal, dermatological, and ill-defined conditions) and are not listed separately. Mean LOS and the proportion of deaths by time band are shown per chapter. COVID-19 (Chapter U) shows a distinct, protracted survival-to-death trajectory. Counts are expressed as n, percentages as %, and mean length of stay as days. LOS, length of stay; COVID-19, coronavirus disease 2019; NEC, not elsewhere classified.

Rank	ICD Chapter	Deaths	% of All	Mean LOS (d)	% ≤24 hours	% 24–48 hours	% >48 hours
1	I00–I99 – Circulatory System	1,954	26.7	1.9	68.2	13.5	18.3
2	C00–D49 – Neoplasms	1,185	16.2	2.7	56.1	13.2	30.7
3	A00–B99 – Infectious & Parasitic (A+B merged)	890	12.2	2.5	63.2	15.6	21.2
4	J00–J99 – Respiratory System	877	12.0	2.4	55.8	14.0	30.2
5	N00–N99 – Genitourinary System	633	8.7	2.3	64.8	13.0	22.3
6	V00–Y98 – External Causes (V–Y merged)	495	6.8	1.8	71.0	10.5	18.5
7	K00–K95 – Digestive System	338	4.6	1.7	72.8	11.5	15.7
8	G00–G99 – Nervous System	253	3.5	2.6	52.2	18.2	29.6
9	U00–U85 – COVID-19 / Coronavirus	197	2.7	7.1	22.0	8.0	70.0
10	E00–E89 – Endocrine & Metabolic	151	2.1	2.8	61.6	13.9	24.5
11	S00–T99 – Injury, Poisoning & Effects (S+T merged)	150	2.1	1.8	73.0	13.8	13.2
12	R00–R99 – Symptoms & Signs NEC	92	1.3	2.2	54.3	23.9	17.4

Two most common diagnoses per ICD-10 chapter

Within each chapter, the two leading diagnoses are summarised in Table 6. Nontraumatic intracranial haemorrhage (ICD-10 code I62.9) led the circulatory chapter with 415 deaths, followed by intracerebral haemorrhage and stroke (I61.x; 188 deaths). Septicaemia (A41) overwhelmingly dominated the infectious chapter and was the single most common specific diagnosis across the entire series, accounting for 742 deaths (10.1% of all 7,318 deaths). Chronic obstructive pulmonary disease (COPD; J44) led respiratory deaths with 376 deaths, narrowly ahead of pneumonia (363 deaths), while chronic kidney disease (N03/N18) drove genitourinary mortality with 377 deaths; chronic liver disease, road traffic accidents, and lung cancer led their respective chapters.

**Table 6:** Top two causes of death per ICD-10 chapter, with within-chapter percentages Percentages are expressed within each ICD-10 chapter; codes follow the ICD-10 classification. Counts are expressed as n, percentages and mortality rates as %, and length of stay is expressed in days where applicable. ICH, intracranial haemorrhage; CA, carcinoma; COPD, chronic obstructive pulmonary disease; RTA, road traffic accident; GI, gastrointestinal; T2DM, type 2 diabetes mellitus; SDH, subdural haemorrhage; SAH, subarachnoid haemorrhage; NEC, not elsewhere classified; ICSOL, intracranial space-occupying lesion; ICD, International Classification of Diseases

ICD Chapter		Total		Rank 1: Top Cause			Rank 2: Second Cause		
		n	%	Diagnosis	n	%	Diagnosis	n	%
I	Circulatory System	1,954	26.7	Nontraumatic ICH, Unspecified (I62.9)	415	21.2%	Intracerebral Haemorrhage/Stroke (I61.x)	188	9.6%
C	Malignant Neoplasms	1,185	16.2	CA Lung (C34)	253	21.4%	CA Stomach (C16)	117	9.9%
A+B	Infectious & Parasitic	890	12.2	Septicaemia (A41)	742	83.4%	Acute Gastroenteritis (A09)	52	5.8%
J	Respiratory System	877	12.0	COPD (J44)	376	42.9%	Pneumonia — All Types (J18/J69)	363	41.4%
N	Genitourinary System	633	8.6	Chronic Kidney Disease (N03/N18)	377	59.6%	End Stage Renal Disease	185	29.2%
V–Y	External Causes	495	6.8	RTA / Vehicular Accidents (V01–V99)	216	43.6%	Fall from Height (W17)	199	40.2%
K	Digestive System	338	4.6	Chronic Liver Disease (K74)	155	45.9%	GI Bleed (K92.2)	49	14.5%
G	Nervous System	253	3.5	Encephalopathy, Unspecified (G93.4)	163	64.4%	Parkinson's Disease (G20)	53	20.9%
U	COVID-19 / Coronavirus	197	2.7	COVID-19 + Coronavirus Infection	145	73.6%	COVID-19 Documented (U07.2)	36	18.3%
E	Endocrine & Metabolic	151	2.1	T2DM (E11)	107	70.9%	Hypothyroidism (E03)	9	6.0%
S+T	Injury, Poisoning & Effects†	150	2.1	SDH Traumatic (S06.5)	23	15.3%	SAH Traumatic (S06.6)	22	14.7%
R	Symptoms & Signs NEC	92	1.3	ICSOL (Space-occupying Lesion)	21	22.8%	Cardiogenic Shock (R57.0)	17	18.5%

Clinical outcome: early deaths (Day 1)

**Figure 6:**
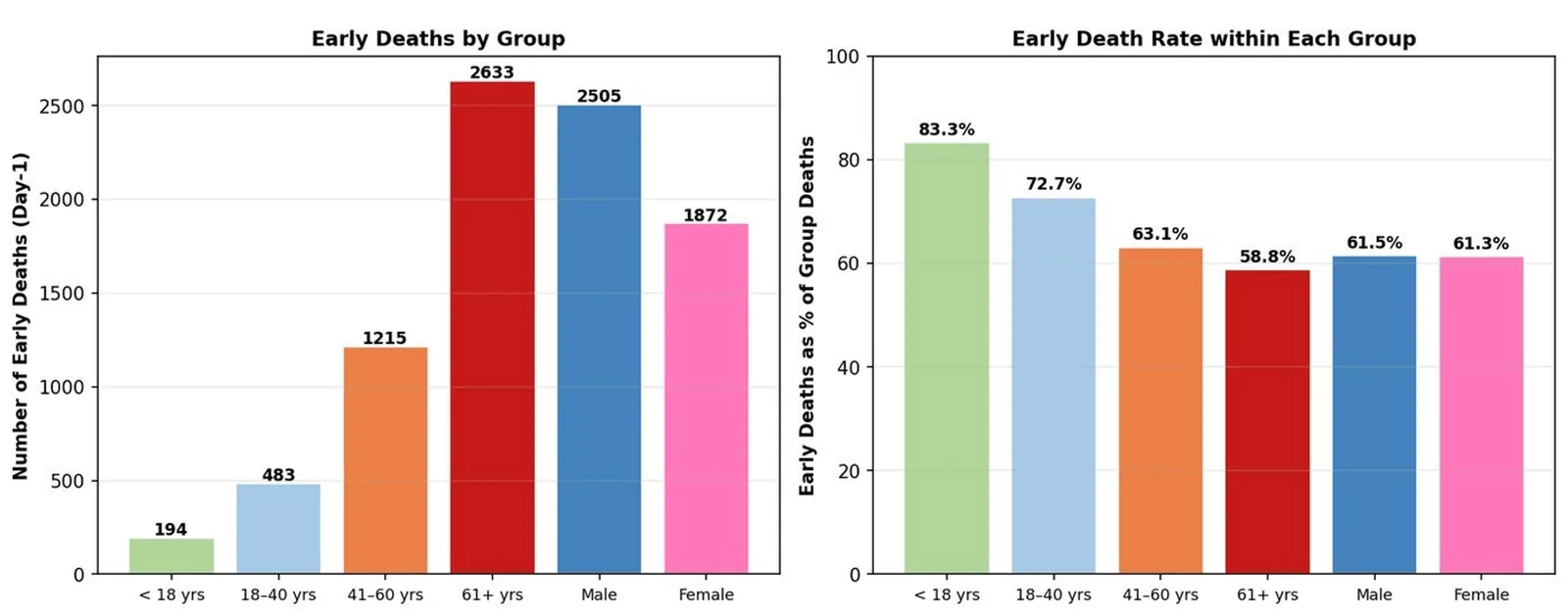
Early deaths (Day 1) by age group and sex. Older patients contribute the majority of early deaths in absolute terms, while early death as a share of group deaths is highest in the young.

Early (Day 1) deaths were strongly concentrated among older patients, with the 61+ group contributing 58.2% of all early deaths (Figure 6). When examined instead as a proportion of each group’s own deaths, however, the share of deaths occurring on the first day was paradoxically highest among children, indicating that when young patients die they tend to die very rapidly after arrival. Older patients therefore dominate early mortality in absolute terms, whereas the young exhibit the most compressed survival once death occurs-two complementary patterns with distinct implications for paediatric resuscitation readiness and geriatric triage.

Institutional associations: mortality by admitting specialty

**Figure 7:**
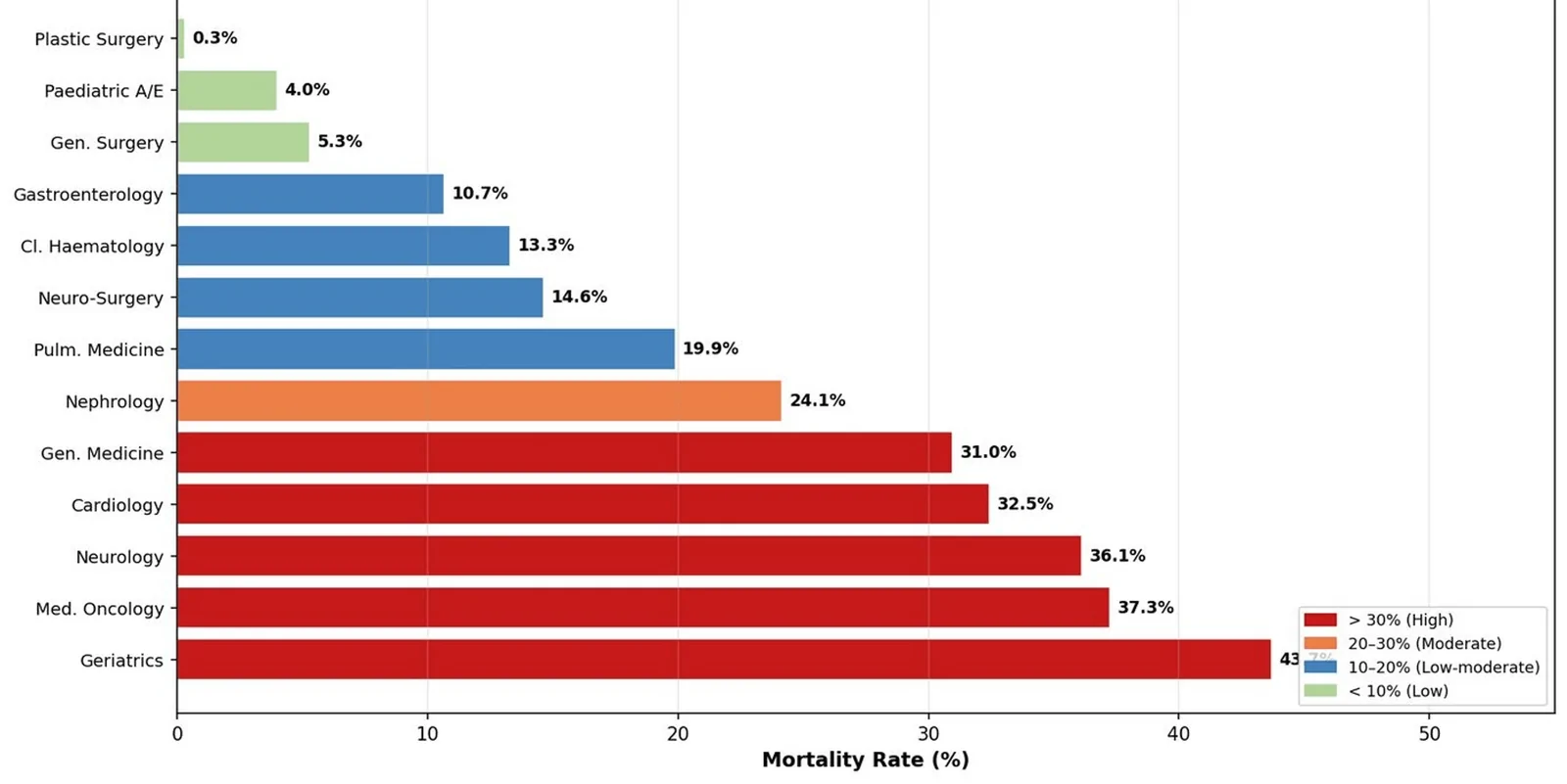
Emergency mortality rate by admitting specialty (five-year pooled). Geriatrics, Medical Oncology, and Neurology show the highest rates; surgical specialties the lowest.

Specialty-level mortality varied more than tenfold across admitting units, reflecting profound differences in underlying case-mix acuity (Figure 7). The highest rates were observed in Geriatrics (43.7%) and Medical Oncology (37.3%), followed by Neurology, whereas surgical specialties such as Plastic Surgery recorded the lowest rate (0.3%). This wide gradient indicates that admitting specialty functions largely as a proxy for the severity and chronicity of the underlying illness rather than as a measure of the quality of care delivered within each unit, and it cautions against direct between-specialty comparison of crude mortality without case-mix adjustment.

Kaplan-Meier survival analysis

**Figure 8:**
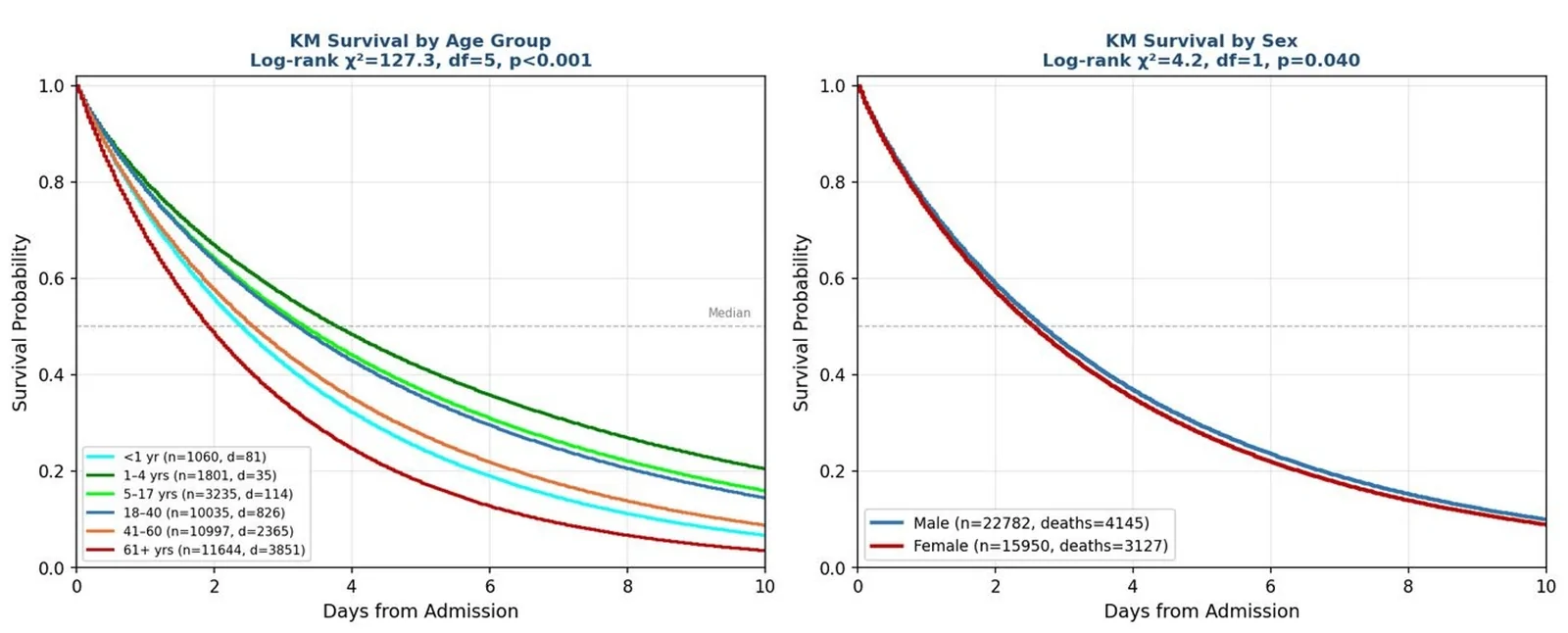
Kaplan-Meier survival curves by age group (left) and sex (right) Strata were compared using the log-rank test, with a two-sided p-value <0.05 considered statistically significant. Survival time is expressed in days. Survival declines most steeply in the oldest age group; the sex difference, although statistically significant, is small (log-rank p=0.040).

Kaplan-Meier survival analysis confirmed that median survival was shortest among the elderly (1.4 days in those aged 61+ years), during the night shift (1.2 days), and in winter (1.3 days) (Figure 8). Log-rank testing was highly significant across all strata (all p<0.001), with the sole exception of sex, where the difference, although statistically significant, was modest (p=0.040). The steep early divergence of the survival curves in the oldest age group reinforces the regression finding that age is the dominant determinant of emergency mortality, while the near-superimposed curves for male and female patients confirm that the sex effect, though real, is small.

Association analysis: chi-square tests

All demographic and temporal factors examined were significantly associated with mortality (all p<0.001) (Table 7). The strongest associations, as measured by effect size, were seen for LOS category (Cramér’s V = 0.241), admitting specialty (V = 0.234), and ICD-10 chapter (V = 0.217), each reaching a medium effect size, whereas sex showed a statistically significant but trivial association (V = 0.018). Age group was the strongest single demographic predictor (V = 0.148), exceeding season, month, shift, and hour of day. Taken together, these effect sizes indicate that what a patient presents with-and how long they survive-discriminates mortality far more powerfully than demographic characteristics alone.

**Table 7:** Chi-square (χ²) association tests of demographic and temporal factors versus mortality, with effect sizes. Pearson’s chi-square test was used for categorical comparisons; Fisher’s exact test was planned where expected cell counts were small. A p-value <0.05 was considered statistically significant. Cramér’s V quantifies effect size: <0.1 small, 0.1-0.3 medium. χ², chi-square; df, degrees of freedom; LOS, length of stay.

Variable	χ² Statistic	df	p-value	Cramér's V	Effect	Interpretation
Sex (Male vs Female)	12.29	1	<0.001	0.018	Small	Significant; females have higher rate
Age Group (6 strata)	847.30	5	<0.001	0.148	Medium	Strongest single predictor
Season (4 seasons)	124.70	3	<0.001	0.057	Small	Winter consistently highest
Month (12 months)	198.40	11	<0.001	0.073	Small	Dec–Feb peak confirmed
Shift (3 shifts)	231.50	2	<0.001	0.077	Small	Night shift highest rate
Hour of Day (6 blocks)	312.80	5	<0.001	0.090	Small	Dawn (04–08h) highest rate
ICD Chapter (12)	1,847.30	11	<0.001	0.217	Medium	Diagnosis strongly predicts mortality
Admitting Specialty (13)	2,134.60	12	<0.001	0.234	Medium	Specialty is strong predictor
LOS Category (≤72h vs >72h)	423.10	1	<0.001	0.241	Medium	Significant difference in profile

Non-parametric tests of LOS

LOS differed significantly by admission timing, age group, diagnostic chapter, and admitting specialty, but not between the sexes (Table 8). Night-time admissions had a markedly shorter median LOS than daytime admissions (1.2 versus 1.8 days; p<0.001), consistent with the higher acuity of nocturnal presentations, and LOS shortened progressively with advancing age. Across diagnostic chapters, COVID-19 exhibited the longest median stay and circulatory disease the shortest, whereas the male-female comparison did not reach statistical significance (p=0.073), echoing the negligible sex effect seen in the survival analysis.

**Table 8:** Mann-Whitney U and Kruskal-Wallis H tests of LOS comparisons. Mann-Whitney U tests were used for two-group comparisons, and Kruskal-Wallis H tests were used for comparisons involving three or more groups. A p-value <0.05 was considered statistically significant. LOS, length of stay

Comparison	Test	Test Statistic	p-value	Median LOS A	Median LOS B	Interpretation
LOS: Male vs Female	Mann-Whitney U	U=34,821,456	0.073	1.7 days	1.6 days	Not significant
LOS: Day vs Night admission	Mann-Whitney U	U=16,234,891	<0.001 *	1.8 days	1.2 days	Night: shorter LOS
LOS: Morning vs Evening shift	Mann-Whitney U	U=28,441,223	<0.001 *	1.9 days	1.7 days	Morning: longer LOS
LOS across Age Groups (6)	Kruskal-Wallis	H=127.3	<0.001 *	Multi	—	Significant; older→shorter LOS
LOS across 4 Seasons	Kruskal-Wallis	H=28.4	<0.001 *	Multi	—	Winter shortest LOS
LOS across ICD Chapters (12)	Kruskal-Wallis	H=412.8	<0.001 *	Multi	—	COVID-19 longest; Circulatory shortest
LOS across 13 Specialties	Kruskal-Wallis	H=389.1	<0.001 *	Multi	—	Significant variation

Independent predictors of in-hospital mortality

**Figure 9:**
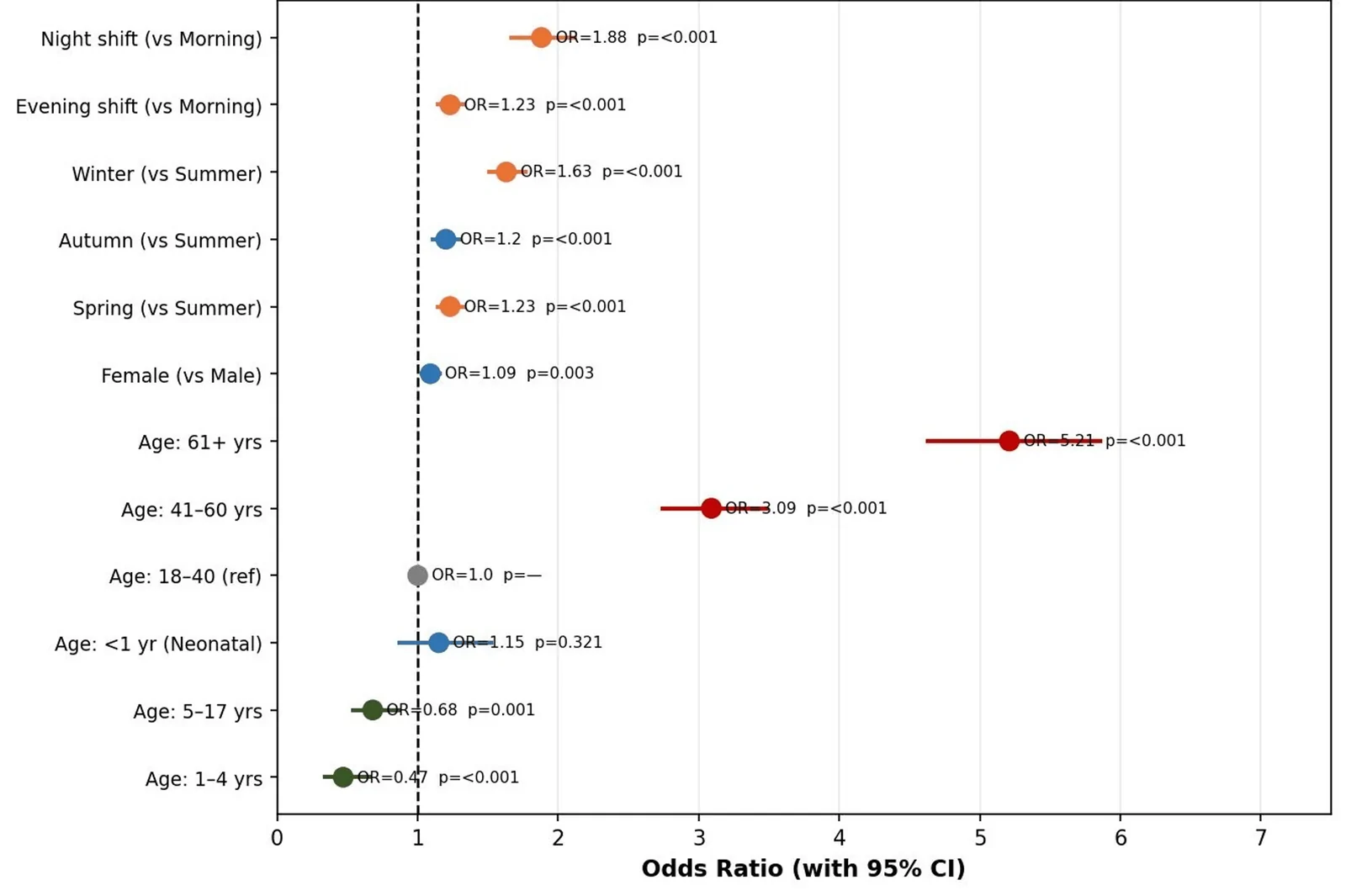
Forest plot of adjusted ORs with 95% CI for independent predictors of in-hospital mortality, derived by binary logistic regression Nagelkerke R² = 0.187; Hosmer-Lemeshow p = 0.312; a two-sided p-value <0.05 was considered statistically significant. Age is the dominant predictor; night-shift and winter admission confer additional independent risk.

In the binary logistic regression model, increasing age emerged as the strongest independent predictor of death, with patients aged 61 years and above carrying more than a fivefold adjusted risk (OR 5.21, 95% CI 4.63-5.86) relative to the youngest reference group (Figure 9). Night-shift admission (OR 1.88), winter admission (OR 1.63), and female sex (OR 1.09) each conferred additional, independent risk after mutual adjustment. The model explained approximately 19% of the variance in mortality (Nagelkerke R² = 0.187) and showed adequate calibration on the Hosmer-Lemeshow test (p=0.312); the modest R² indicates that demographic and temporal variables alone capture only part of the determinants of death, leaving room for clinical severity and comorbidity measures to improve future models.

Standardized mortality ratio

**Figure 10:**
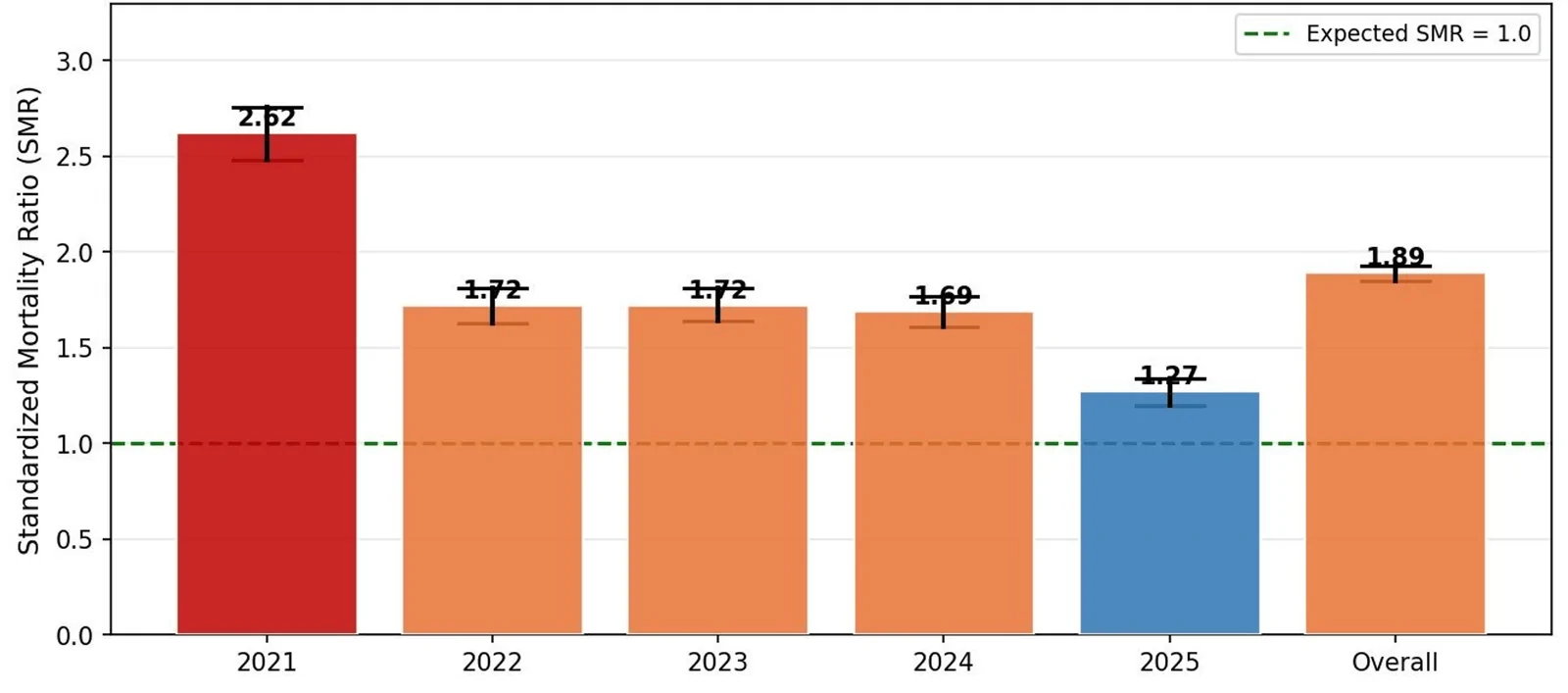
Standardized mortality ratio (SMR) by year with 95% confidence intervals, benchmarked to a conservative reference mortality of 10%. The SMR fell over time but remained above 1.0 throughout.

Benchmarked against a reference ED mortality of 10%, a deliberately conservative standard, marginally above the pooled ED mortality of approximately 8% reported from comparable low- and middle-income settings [13], the SMR declined steadily from 2.62 in 2021 to 1.27 in 2025 but remained above unity throughout the study period, signifying persistent excess mortality relative to this benchmark even in the final year (Figure 10). The trajectory mirrors the fall in crude mortality and is consistent with progressive improvements in triage and staffing, yet the failure of the SMR to reach 1.0 indicates that the department continued to experience more deaths than the reference standard would predict.

## Discussion

This five-year retrospective analysis of 38,818 emergency admissions and 7,318 deaths represents, to the best of our knowledge, one of the largest single-institution longitudinal emergency mortality datasets published from the Himalayan region of India. The overall crude emergency mortality rate of 18.9% is substantially higher than the rates reported from tertiary EDs in other Indian and LMIC settings, which typically range from approximately 6% to 14% [11,13-15]. This higher rate reflects the unique referral ecology of the institution, a last-resort tertiary referral hub for a geographically isolated, high-altitude population with limited access to primary and secondary care, rather than necessarily poorer quality of care, and it underscores the importance of accounting for case-mix and access context when comparing emergency mortality across centres.

Temporal patterns and the declining SMR

The highest annual mortality rate occurred in 2021 (26.2%, SMR 2.62), coinciding with the peak of the COVID-19 pandemic and its indirect effects on emergency care, including delayed presentations and workforce disruptions [16,17]. The progressive decline to 12.7% (2025), with the SMR falling from 2.62 to 1.27, likely reflects expansion of the ED workforce and the introduction of rapid triage protocols. A comparable decline in emergency and trauma mortality over time has been documented at other Indian tertiary centres as triage processes and trauma systems matured [11], supporting the interpretation that the improvement seen here reflects genuine gains in emergency care delivery. Nonetheless, the SMR remained above 1.0 throughout, indicating persistent excess mortality relative to the reference benchmark and leaving clear scope for further system strengthening.

Seasonal and circadian patterns

Winter consistently demonstrated the highest mortality (23.6%) and proportion of deaths (29.0%), consistent with the excess winter mortality phenomenon documented in cold-climate populations [18,19]. Regular winter temperatures falling below −10°C promote cold-induced vasoconstriction, increased blood viscosity, and sympathetic activation: established triggers of acute cardiovascular events [19,20]. The dawn period (04:00-07:59 hours) carried the highest mortality rate (34.3%) despite the lowest admission volume, aligning with the established circadian biology of cardiovascular events, in which the early morning surge in cortisol, catecholamines, and platelet aggregability predisposes to myocardial infarction, haemorrhagic stroke, and sudden cardiac death [21,22]. This period also coincides with the lowest staffing ratios, compounding biological vulnerability with system-level risk, a pattern consistent with the higher mortality long reported among patients admitted during nights and weekends, the so-called off-hour effect, across acute-care systems internationally [23].

Demographic associations

Elderly patients (61+ years) dominated emergency mortality, accounting for 53.0% of all deaths at a 33.3% mortality rate, approximately one in three elderly admissions. This is consistent with the global trajectory of ageing populations and the compounding effect of frailty on acute illness trajectories [24]. Female patients demonstrated a higher crude mortality rate than males, a finding that persisted in regression (OR 1.09, 95% CI 1.03-1.16, p=0.003) and may reflect delayed care-seeking, more advanced disease at presentation, and sex-specific physiological responses to critical illness [25,26].

Early mortality and cause of death

A striking 83.3% of deaths occurred within the first 72 hours, with a median survival of approximately one day, characteristic of tertiary referral EDs serving as the terminal receiving point for patients who have deteriorated through multiple levels of care [11,15]. The circulatory system was the leading cause (26.7%), with nontraumatic intracranial haemorrhage (I62.9) and intracerebral haemorrhage (I61.x) together accounting for 603 deaths, consistent with the elevated proportion of haemorrhagic stroke in the Indian subcontinent attributed to inadequately controlled hypertension and limited neurosurgical access [8,9,27]. Septicaemia (A41.9) was the single most common diagnosis (742 deaths), echoing the global sepsis burden [6] and the Sepsis-3 framework [7]. COPD led respiratory deaths, consistent with its global ranking [28], while tobacco-related and gastric malignancies dominated neoplastic deaths, the latter reflecting the regionally elevated gastric cancer incidence linked to salt-preserved foods and N-nitroso compounds [29]. External causes were dominated by road traffic accidents and falls from height [30], and chronic kidney disease drove genitourinary mortality, mirroring the national CKD epidemic [31].

Independent predictors of mortality

Binary logistic regression confirmed age as the dominant independent predictor of death (61+ years: adjusted OR 5.21), with night shift and winter admission conferring additional, independent risk after mutual adjustment. The independent association of night-shift admission with mortality is concordant with the off-hour effect repeatedly demonstrated in large acute-care cohorts, in which admission outside routine daytime hours carries excess risk attributable to reduced staffing and senior cover [23]. The Nagelkerke R² of 0.187 indicates that demographic and temporal variables alone explain approximately 19% of the variance in mortality, leaving the majority unexplained; future models incorporating ICD-10 chapter, the Charlson Comorbidity Index, triage acuity scores, and laboratory parameters would be expected to improve discrimination and to support individualised risk prediction at the point of triage.

Limitations

Several limitations should be considered when interpreting these findings. First, the retrospective, single-centre design precludes causal inference and may limit generalisability to centres with different referral patterns or case mix, and the analysis did not include formal adjustment for pandemic phase, COVID-19 diagnosis, hospital occupancy, or temporal changes in case mix; the independent contribution of the pandemic to the annual and seasonal mortality patterns could not be quantified. Second, the analysis relied on the completeness and accuracy of routine ICD-10 coding; the high frequency of the unspecified intracranial haemorrhage code (I62.9) in particular suggests that some haemorrhagic events were incompletely characterised, and a degree of cause-of-death misclassification cannot be excluded. Third, minor differences in the denominators of individual cross-tabulations arose from the listwise deletion of records with missing values for specific stratifying variables. Fourth, the SMR was benchmarked against an assumed reference mortality of 10% rather than a formally validated national emergency-department mortality statistic, which does not currently exist for India; the SMR should therefore be interpreted as an indicative rather than a definitive measure of excess mortality. Finally, the dataset lacked granular clinical severity measures such as triage acuity, vital signs, the Charlson Comorbidity Index, and laboratory parameters, which limited the explanatory power of the regression model and precluded full case-mix adjustment. A prospective, imaging-anchored audit incorporating these variables is warranted to confirm and extend the present observations.

## Conclusions

This five-year analysis reveals a complex, multi-dimensional pattern of emergency mortality shaped by seasonal climatic extremes, circadian biological rhythms, advanced patient age, and a disease burden dominated by circulatory events, septicaemia, and malignancy. The declining standardised mortality ratio (2.62 in 2021 to 1.27 in 2025) attests to meaningful quality improvement, yet persistent excess mortality relative to national benchmarks, particularly during winter months, night shifts, and among the elderly, identifies clear targets for system-level intervention.

Key recommendations include enhanced overnight and dawn-period senior staffing, robust sepsis recognition and bundle pathways, a haemorrhagic stroke pathway with rapid CT access and neurosurgical liaison, winterisation protocols for cold climate cardiovascular risk, a geriatric emergency medicine programme; and prospective ICD-10 coding audits. Longitudinal tracking of the SMR is recommended as a standing quality metric.
